# C‐Reactive Protein to Lymphocyte Ratio (CLR) and Lactate Dehydrogenase to Albumin Ratio (LAR) as Prognostic Biomarkers in Acral Melanoma: Association With Tertiary Lymphoid Structures and Immune Cell Infiltration

**DOI:** 10.1002/cam4.71078

**Published:** 2025-07-26

**Authors:** Donglin Kang, Xinyu Su, Jiayu Wang, Lianjun Zhao, Rong Huang, Zhengyun Zou

**Affiliations:** ^1^ Department of the Comprehensive Cancer Center Nanjing Drum Tower Hospital Clinical College of Nanjing Medical University Nanjing China; ^2^ Department of the Comprehensive Cancer Center Nanjing Drum Tower Hospital, Affiliated Hospital of Medical School, Nanjing University Nanjing China; ^3^ Department of Oncology Nanjing Drum Tower Hospital Clinical College of Nanjing University of Chinese Medicine Nanjing China

**Keywords:** acral melanoma, blood biomarkers, tertiary lymphoid structure, tumor microenvironment

## Abstract

**Objective:**

To investigate the correlation between peripheral blood CRP‐to‐lymphocyte ratio (CLR) and lactate dehydrogenase‐to‐albumin ratio (LAR) levels, prognosis, and the tumor microenvironment (TME) in patients with acral melanoma (AM), with a focus on tertiary lymphoid structures (TLS) and immune cell infiltration.

**Methods:**

A retrospective cohort of 36 patients with acral melanoma was included. Clinical data and hematological parameters were collected. The maturity of TLS, the proportion of immune cells, and their spatial distribution within the TME were assessed using H&E staining and multiplex immunofluorescence. Kaplan–Meier survival curves and Cox regression models were employed to examine the association between these indicators and patient survival. Non‐parametric tests and Spearman's correlation analysis were used to compare CLR and LAR levels across patients with different TLS maturity and immune infiltration statuses.

**Results:**

Elevated levels of CLR and LAR in the blood are associated with poorer prognosis in patients with acral melanoma. The levels of CLR and LAR vary across patients with TLS of different maturities, and these levels decrease as TLS maturity increases. Additionally, CLR and LAR levels are significantly correlated with the proportions of CD4+ and CD8+ T cells in TME, with higher levels of CLR and LAR being linked to reduced immune cell infiltration.

**Conclusion:**

Elevated CLR and LAR levels are associated with poorer prognosis in AM patients, and this relationship is closely linked to the maturity of TLS and the extent of immune cell infiltration within the TME.

## 1 Introduction

Melanoma is one of the most aggressive malignant tumors in the world, with an estimated 330,000 new cases and 58,000 deaths in 2022 [1]. While 7.5% of all melanoma cases occur in Asia, the continent accounts for approximately 22.4% of global melanoma‐related deaths. This discrepancy is partly attributed to the unique subtype of melanoma prevalent in Asian populations: acral melanoma (AM). Unlike other forms of melanoma, AM primarily affects areas that are not exposed to ultraviolet (UV) radiation, such as the palms, soles, and nails. In recent years, immunotherapy, particularly immune checkpoint inhibitors, has shown substantial efficacy in improving overall survival in patients with cutaneous melanoma. However, these treatments have proven less effective for those with AM [2, 3]. Tumor microenvironment (TME) is a dynamic and complex milieu surrounding the tumor, composed of extracellular matrix and immune cells. Research targeting the TME can provide deeper insights into the pathogenesis and progression of AM, as well as the mechanisms underlying immune evasion. Tertiary lymphoid structures (TLS) within the TME are also considered to be closely associated with favorable responses to anti‐tumor immunotherapy [4, 5]. Assessing the TME presents significant challenges, particularly during or after immunotherapy, due to its dynamic nature. Consequently, there is an increasing need for accessible biomarkers to effectively monitor immune responses in these settings.

The pathogenesis and progression of cancer are strongly correlated with systemic inflammatory responses and metabolic alterations [6, 7, 8]. Extensive research has demonstrated that blood biomarkers, such as inflammatory and metabolic indicators, can effectively predict patient prognosis [9, 10, 11]. Lymphocyte count and C‐reactive protein (CRP) levels are routinely measured, with the CRP‐to‐lymphocyte ratio (CLR) serving as a prognostic marker in cancer. Lactate dehydrogenase (LDH) is a key enzyme in anaerobic glycolysis, catalyzing the conversion of pyruvate to lactate under hypoxia. The immune evasion driven by lactate accumulation is one of the key factors that facilitate tumor growth, progression, and metastasis [12, 13]. Serum albumin, an important indicator of nutritional status and liver function, is closely linked to cancer prognosis. Hypoalbuminemia is frequently observed in patients with advanced‐stage cancer, often indicative of impaired liver function or malnutrition, and correlates with poor clinical outcomes. Notably, the lactate dehydrogenase‐to‐albumin ratio (LAR) has emerged as a reliable independent prognostic factor in several cancers, including colorectal cancer [14], bladder cancer [15], nasopharyngeal carcinoma [16], breast cancer [17] and esophageal cancer [18], with higher ratios correlating with worse prognosis and diminished survival outcomes. However, studies exploring the relationship between these markers, TLS, and TME in patients with AM remain limited.

In this study, we retrospectively collected clinical data and tissue samples from AM patients and evaluated the correlation between serum levels of CLR and LAR and patient prognosis. Additionally, we further analyzed their association with the status of TLS and immune cell infiltration within the tumor microenvironment.

## 2 Materials and Methods

### 2.1 Patients

This retrospective analysis included 36 patients diagnosed with acral melanoma at Nanjing Drum Tower Hospital between January 2013 and December 2021. Inclusion criteria were: (1) patients who underwent surgical treatment and were subsequently confirmed by postoperative pathological examination to have acral melanoma, and (2) those with complete preoperative laboratory data and available follow‐up information. Exclusion criteria comprised: (1) patients who received preoperative antitumor therapy, (2) those with concurrent malignancies, (3) individuals with hematological or autoimmune disorders, and (4) patients with recent acute or chronic infectious diseases or other conditions that could potentially affect biomarker levels. The study adhered to the principles of the Declaration of Helsinki and was approved by the Ethics Committee of Nanjing Drum Tower Hospital (Approval No. 2024‐282‐01). Written informed consent was obtained from all participants prior to their inclusion in the study.

### 2.2 Data Collection

Baseline data, including age, sex, clinical stage, postoperative pathology, laboratory test results, and tissue wax blocks, were systematically collected. Clinical staging was based on the eighth edition of the AJCC Cancer Staging System. Blood tests assessed white blood cell (WBC) count, lymphocyte count, albumin, C‐reactive protein (CRP), and LDH levels. The C‐reactive protein to lymphocyte ratio (CLR) was calculated as CRP divided by lymphocyte count, and the lactate dehydrogenase to albumin ratio (LAR) as LDH divided by albumin. Long‐term prognosis was evaluated using overall survival (OS). Data were collected from medical records and follow‐up telephone interviews. Formalin‐fixed paraffin‐embedded (FFPE) tissue blocks were retrieved from the Department of Pathology at Nanjing Drum Tower Hospital. All samples included in this study were primary tumor specimens obtained from postoperative paraffin‐embedded tissue sections of the patients' tumor primary sites.

### 2.3 Immunohistochemistry and Image Analysis

**FIGURE 1:**
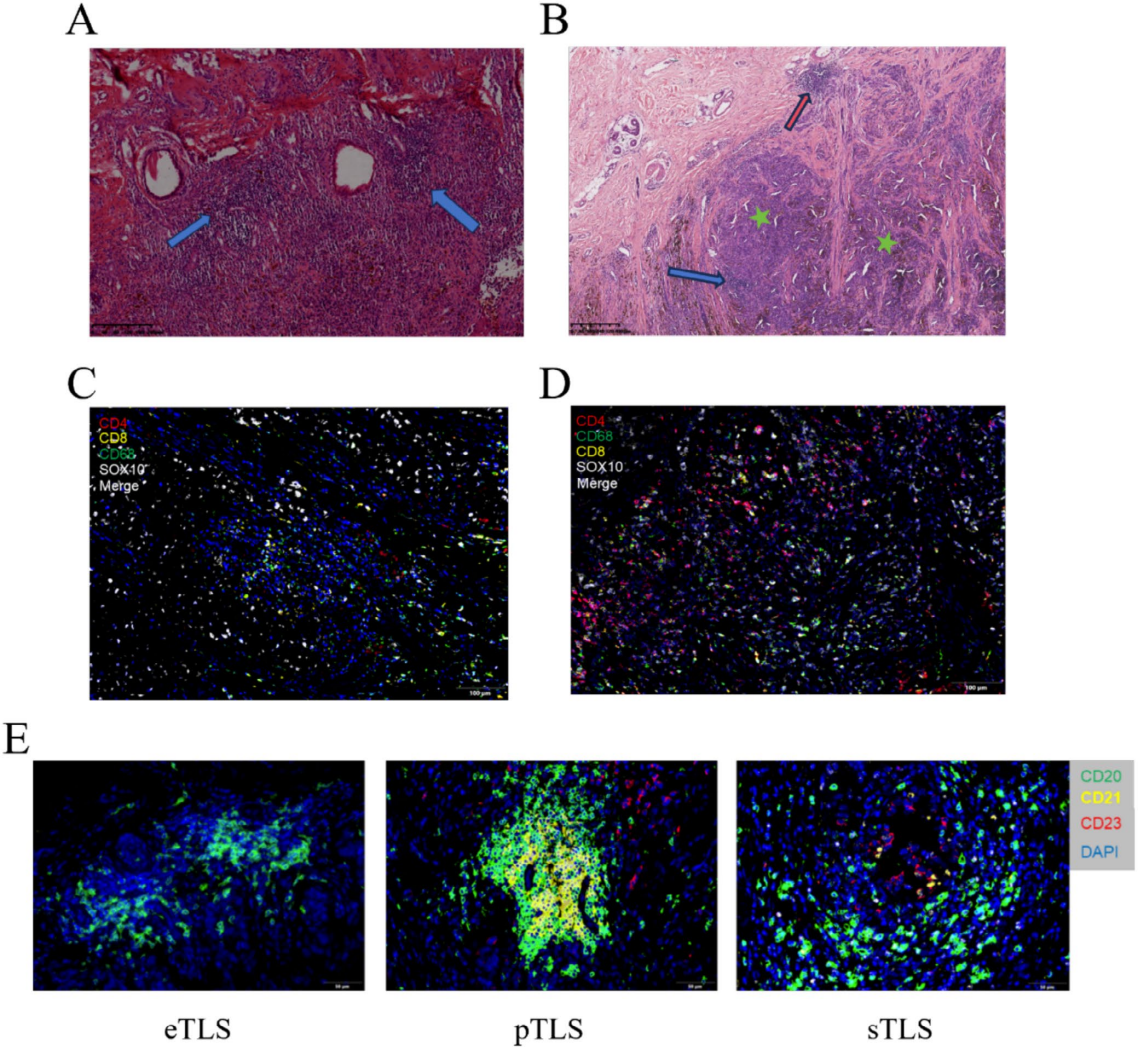
Characterization of TLS and TME in melanoma. (A) H&E staining image of TLSs showing lymphocyte aggregation (≥ 50 lymphocytes, indicated by blue arrows). (B) Intratumoral TLS (blue arrows), surrounded by tumor cells (blue stars); Peritumoral TLS (red arrows), located at the invasive tumor margin. (C) Representative mIF staining of TLS cellular components. (D) Representative mIF staining of TME cellular components. (E) Representative images of TLSs at different maturation stages in tumor specimens. eTLS, CD20^+^ lymphocyte aggregates without follicular organization; pTLS, CD20^+^ aggregates containing CD21^+^ FDC but lacking CD23^+^ GC; sTLS, CD20^+^ aggregates with CD21^+^ FDC and CD23^+^ GC.

For the multiplex immunofluorescence (mIF) analysis, we utilized the PANO 5‐plex IHC kit, incorporating Tyramide Signal Amplification (TSA), in accordance with the manufacturer's protocol (Panovue, China). Tumor samples, fixed in formalin and embedded in paraffin (FFPE), were sectioned into 4 μm slices for mIF staining. Multispectral imaging of the stained slides was performed using the PanoVIEW VS200 System (Panovue, China), with both acidic and alkaline antigen retrieval solutions (Panovue, China) applied where necessary. The staining procedure involved two antibody panels, each containing 3 to 4 antibodies. The composition of these panels is as follows: Panel 1: TLS Maturation: CD20 (1:400, Proteintech, USA, Cat. no. 60271–1‐Ig) with Opal520; CD21 (1:100, Abcam, USA, Cat. no. ab227668) with Opal570; CD23 (1:100, Proteintech, USA, Cat. no. 60208–1‐Ig) with Opal650; and spectral DAPI (Beyotime Biotechnology, China, Cat. no. C10002) (Figure 1B,C). Panel 2: TME: CD4 (1:400, Proteintech, USA, Cat. no. 76069‐1‐Ig) with Opal520; CD8 (1:200, Proteintech, USA, Cat. no. 66868‐1‐Ig) with Opal570; CD68 (1:2000, Proteintech, USA, Cat. no. 66231‐2‐Ig) with Opal650; SOX10 (1:400, Proteintech, USA, Cat. no. 66786‐1‐Ig) with Opal480; and spectral DAPI (Beyotime Biotechnology, China, Cat. no. C10002) (Figure 1D). Multispectral images were processed using spectral unmixing techniques, applying single‐stain positive control images within the InForm software (Akoya Biosciences, USA). To quantify the immune cell distribution within the TME, the percentage of positively stained immune cells was calculated relative to the total number of nucleated cells.

### 2.4 Evaluation of TLS in Pathological Sections

TLS were independently identified in a double‐blind manner by two experienced pathologists based on hematoxylin–eosin (H&E) staining and mIF staining [19, 20]. In H&E‐stained sections, TLS were defined as lymphocytic aggregates containing ≥ 50 lymphocytes within the lesion area (Figure 1A) In mIF staining, TLS were characterized as unencapsulated aggregates of CD20^+^ B cells adjacent to CD4^+^ and CD8^+^ T cell zones (Figure 1C). TLS were categorized based on their location within the tumor tissue as intratumoral TLS and peritumoral TLS at the invasive margin adjacent to normal tissue [21] (Figure 1B). To evaluate TLS maturation, CD21 and CD23 status in mIF staining were used as markers to distinguish follicular dendritic cells (FDC) and germinal centers (GC), respectively [19, 22, 23, 24]. TLS with only CD20^+^ B cells and aggregates of CD4^+^ and CD8^+^ T cells, but lacking CD21^+^ FDC and CD23^+^ GC, were classified as early stage TLS (eTLS). TLS containing CD21^+^ FDC but lacking CD23^+^ GC were categorized as primary follicle‐like TLS (pTLS). TLS with both CD21^+^ FDC and CD23+ GC were defined as secondary follicle‐like TLS (sTLS) (Figure 1E).

### 2.5 Statistics

Data were analyzed using R 4.2.3, SPSS 27, and GraphPad Prism 9.5. Chi‐square or Fisher's exact tests were used for categorical variables, while continuous variables were compared using an independent t‐test (for normally distributed data with equal variances) or Mann–Whitney U test. Spearman's correlation assessed the relationship between CLR, LAR, and immune cell infiltration in the TME. ROC curve analysis evaluated CLR and LAR's diagnostic performance for patient survival. OS was analyzed with Kaplan–Meier and log‐rank tests, and Cox regression identified OS‐associated factors. Statistical significance was set at *p* < 0.05 (two‐tailed).

## 3 Result

### 3.1 Demographics and Clinical Characteristics

In this study, a total of 36 patients with AM were included, comprising 17 males (47.22%) and 19 females (52.78%), with an average age of 66.14 ± 10.18 years. According to the TNM staging system, 4 patients (11.11%) were classified as stage I, 17 patients (47.22%) as stage II, and 15 patients (41.67%) as stage III. In terms of TLS characteristics, 33 cases (91.67%) exhibited TLS, of which 24 cases (66.67%) showed intratumoral TLS, while 30 patients (83.33%) presented peritumoral TLS. Further laboratory results are provided in Table 1.

**TABLE 1:** Demographic and Clinical Characteristics of the Patients.

Variables	Total (*n* = 36)
Age (years), Mean ± SD	66.14 ± 10.18
Sex, *n* (%)	
Male	17 (47.22%)
Female	19 (52.78%)
Stage, *n* (%)	
I	4 (11.11%)
II	17 (47.22%)
III	15 (41.67%)
TLS, *n* (%)	
Without	3 (8.33%)
With	33 (91.67%)
Intratumoral TLS, *n* (%)	
Without	12 (33.33%)
With	24 (66.67%)
Peritumoral TLS, *n* (%)	
Without	6 (16.67%)
With	30 (83.33%)
WBC (×10^9^/L), M (Q_1_, Q_3_)	5.80 (4.77, 6.72)
Lymphocytes (×10^9^/L), M (Q_1_, Q_3_)	1.75 (1.48, 1.92)
CRP (mg/L), M (Q_1_, Q_3_)	2.15 (0.75, 3.32)
LDH (U/L), M (Q_1_, Q_3_)	167.50 (148.00, 177.25)
Albumins (g/L), M (Q_1_, Q_3_)	40.60 (39.35, 41.70)
LAR, M (Q_1_, Q_3_)	4.07 (3.62, 4.42)
CLR, M (Q_1_, Q_3_)	1.18 (0.42, 2.22)

Abbreviations: M, median; Q_1_, 1st Quartile; Q_3_, 3st Quartile; SD, standard deviation.

### 3.2 Analysis of OS

The optimal cutoff values for predicting OS based on age, CLR, and LAR were determined by ROC curve analysis. According to Kaplan–Meier analysis, the overall survival in the younger age group was significantly better than that in the older age group (*p* = 0.008, HR = 0.296). Patients in the low LAR group had significantly better OS compared to those in the high LAR group (*p* = 0.031, HR = 0.227). Similarly, the low CLR group demonstrated significantly better OS compared to the high CLR group (*p* = 0.002, HR = 0.201). In the univariate Cox regression analysis, age, sex, stage, CLR, and LAR were included as covariates. The results demonstrated that age, CLR, and LAR were significant predictors of OS. A subsequent multivariate Cox regression analysis showed that age (*p* = 0.022, HR =3.22), CLR (*p* = 0.009, HR = 4.98), and LAR (*p* = 0.015, HR = 6.81) were independent prognostic factors for OS.

**FIGURE 2:**
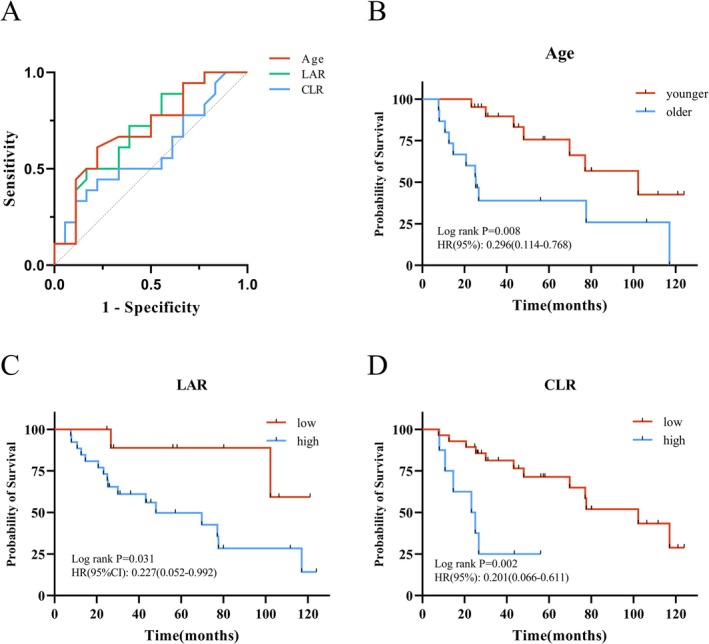
Cutoff values and Kaplan‐Meier survival curves for overall survival (OS). (A) The cutoff values for Age, CLR, and LAR were determined using the ROC curve analysis, with values of 69.5 years, 2.72, and 3.73, respectively; (B–D) Kaplan‐Meier survival curves for overall survival based on the cutoff values mentioned above.

The optimal cutoff values for predicting OS were determined using ROC curve analysis (Figure 2A). Kaplan–Meier analysis demonstrated that OS in the older age group was significantly worse than in the younger group (*p* = 0.008, HR = 0.296). Patients with high LAR had significantly poorer OS compared to those with low LAR (*p* = 0.031, HR = 0.227), and similarly, the high CLR group had significantly worse OS than the low CLR group (*p* = 0.002, HR = 0.201) (Figure 2B–D). Both univariate and multivariate Cox regression analyses identified age (*p* = 0.022, HR = 3.22), CLR (*p* = 0.009, HR = 4.98), and LAR (*p* = 0.015, HR = 6.81) as independent prognostic factors for OS (Table 2).

**TABLE 2:** Univariate and multivariate cox regression analysis of OS.

Variables	Univariate analysis					Multivariate analysis				
	β	S.E	*Z*	*p*	HR (95% CI)	β	S.E	*Z*	*p*	HR (95% CI)
Age (years)										
≤ 69.5					1.00 (Reference)					1.00 (Reference)
> 69.5	1.22	0.49	2.50	**0.012**	3.38 (1.30 ~ 8.77)	1.17	0.51	2.28	**0.022**	3.22 (1.18 ~ 8.77)
Sex, *n* (%)										
Male					1.00 (Reference)					
Female	0.08	0.5	0.16	0.869	1.09 (0.41 ~ 2.89)					
Stage										
I					1.00 (Reference)					
II	1.18	1.11	1.07	0.286	3.25 (0.37 ~ 28.43)					
III	1.89	1.07	1.78	0.075	6.65 (0.82 ~ 53.65)					
CLR										
≤ 2.72					1.00 (Reference)					1.00 (Reference)
> 2.72	1.61	0.57	2.83	**0.005**	4.98 (1.64 ~ 15.17)	1.61	0.61	2.62	**0.009**	4.98 (1.50 ~ 16.55)
LAR										
≤ 3.73					1.00 (Reference)					1.00 (Reference)
> 3.73	1.48	0.75	1.97	**0.049**	4.41 (1.01 ~ 19.28)	1.92	0.79	2.44	**0.015**	6.81 (1.46 ~ 31.90)

Abbreviations: CI, confidence interval; HR, hazard ratio. *Note*: The bold values highlight statistically significant results (*p* < 0.05).

### 3.3 Comparison of Clinical Characteristics and Laboratory Parameters at Different Stages of TLS Maturity

**FIGURE 3:**
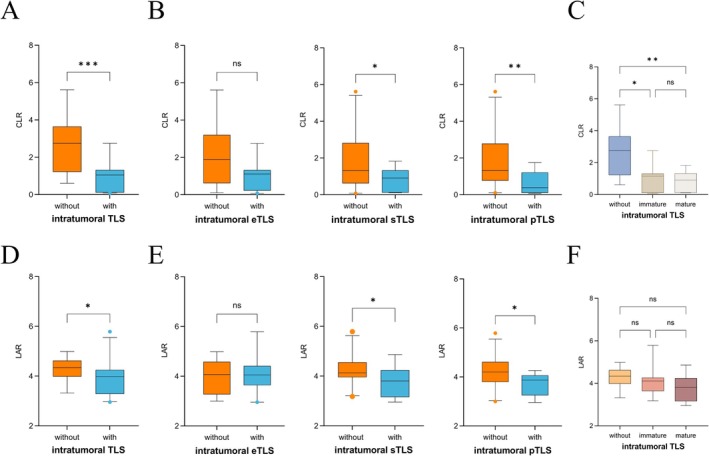
Correlation between blood parameters and TLS status. (A) The CLR is significantly reduced in the tumor‐infiltrating TLS group. (B) Intra‐group comparison of CLR across TLS subsets of different maturities. (C) Inter‐group comparison of CLR across TLS subsets of different maturities. (D) The LAR is significantly reduced in the tumor‐infiltrating TLS group. (E) Intra‐group comparison of LAR across TLS subsets of different maturities. (F) Inter‐group comparison of LAR across TLS subsets of different maturities (*p* < 0.05*, *p* < 0.01**, *p* ≤ 0.001***, ns: no significant difference).

Next, we analyzed the correlation between blood‐based prognostic factors and the maturation of TLS. Our results indicated that patients without intratumoral TLS had significantly higher levels of CRP and CLR compared to those with intratumoral TLS (all *p* < 0.001) (Figure 3A,D). Based on TLS maturity, patients were then classified into eTLS, pTLS, and sTLS groups. Those without eTLS had lower WBC (*p* = 0.046) and differed in clinical stage (*p* = 0.045). Patients without pTLS had higher CRP (*p* = 0.016), LAR (*p* = 0.049), and CLR (*p* = 0.008), with lower lymphocyte count (*p* = 0.046) and albumin (*p* = 0.02). The sTLS group showed higher CRP (*p* = 0.017), LAR (*p* = 0.032), and CLR (*p* = 0.031) (Figure 3B,E and Table 3). When comparing CLR and LAR across groups, patients were classified into those without TLS, immature TLS (including eTLS and pTLS), and mature TLS (sTLS). Both CLR and LAR exhibited a decreasing trend. Compared to the without TLS group, CLR showed a significant difference across the groups, while no statistically significant difference was observed in the LAR groups (Figure 3C,F). However, no statistically significant differences were observed in CLR and LAR within the peritumoral TLS group (Table S1). Furthermore, since nearly all patients exhibited peritumoral TLS within their tumors, we limited our analysis to the intratumoral TLS and peritumoral TLS groups, assessing the status of intratumoral TLS.

**TABLE 3:** Comparison of clinical characteristics and laboratory parameters at different stages of intratumoral TLS maturity.

Variables	Intratumoral eTLS			Intratumoral pTLS			Intratumoral sTLS			Intratumoral TLS		
	Without (*n* = 17)	With (*n* = 19)	*p*	Without (*n* = 25)	With (*n* = 11)	*p*	Without (*n* = 23)	With (*n* = 13)	*p*	Without (*n* = 12)	With (*n* = 24)	*p*
Age (years), Mean ± SD	66.88 ± 12.58	65.47 ± 7.73	0.685	67.80 ± 9.40	62.36 ± 11.31	0.142	67.09 ± 10.70	64.46 ± 9.36	0.465	68.42 ± 11.37	65.00 ± 9.58	0.35
Sex, *n* (%)			1			0.721			1			1
Male	8 (47.06)	9 (47.37)		11 (44.00)	6 (54.55)		11 (47.83)	6 (46.15)		6 (50.00)	11 (45.83)	
Female	9 (52.94)	10 (52.63)		14 (56.00)	5 (45.45)		12 (52.17)	7 (53.85)		6 (50.00)	13 (54.17)	
Stage, *n* (%)			**0.045**			0.223			0.89			0.184
I	2 (11.76)	2 (10.53)		3 (12.00)	1 (9.09)		2 (8.70)	2 (15.38)		0 (0.00)	4 (16.67)	
II	5 (29.41)	12 (63.16)		9 (36.00)	8 (72.73)		11 (47.83)	6 (46.15)		5 (41.67)	12 (50.00)	
III	10 (58.82)	5 (26.32)		13 (52.00)	2 (18.18)		10 (43.48)	5 (38.46)		7 (58.33)	8 (33.33)	
WBC, M (Q_1_, Q_3_)	5.00 (4.60, 5.80)	6.10 (5.30, 7.95)	**0.046**	5.80 (4.60, 6.10)	6.10 (5.15, 8.90)	0.068	5.80 (4.75, 6.85)	6.00 (4.80, 6.10)	1	4.95 (4.57, 5.85)	6.05 (4.88, 7.80)	0.111
Lymphocytes (×109/L), M (Q_1_, Q_3_)	1.70 (1.50, 1.90)	1.80 (1.45, 2.05)	0.382	1.60 (1.40, 1.90)	1.90 (1.75, 2.20)	**0.046**	1.60 (1.45, 1.90)	1.90 (1.50, 2.00)	0.372	1.55 (1.45, 1.80)	1.85 (1.48, 2.02)	0.147
CRP (mg/L), M (Q_1_, Q_3_)	3.10 (1.10, 4.60)	2.10 (0.40, 2.40)	0.111	2.50 (1.80, 4.40)	0.80 (0.20, 2.20)	**0.016**	3.10 (1.45, 4.45)	2.00 (0.20, 2.20)	**0.017**	3.95 (2.83, 5.32)	2.00 (0.20, 2.30)	**0.001**
LDH (U/L), M (Q_1_, Q_3_)	169.00 (134.00, 176.00)	166.00 (152.50, 179.00)	0.558	172.00 (151.00, 179.00)	165.00 (140.50, 174.00)	0.257	173.00 (162.50, 178.00)	156.00 (132.00, 174.00)	0.093	174.00 (168.25, 177.50)	163.00 (133.50, 176.50)	0.164
Albumins (g/L), M (Q_1_, Q_3_)	40.60 (38.50, 40.80)	40.70 (39.45, 41.80)	0.456	39.80 (38.50, 40.80)	41.30 (40.60, 42.70)	**0.036**	40.10 (38.60, 41.70)	40.70 (39.50, 41.60)	0.468	39.85 (37.80, 41.25)	40.65 (39.50, 41.70)	0.260
LAR, M (Q_1_, Q_3_)	4.07 (3.32, 4.55)	4.05 (3.73, 4.34)	0.975	4.20 (3.95, 4.60)	3.87 (3.40, 4.06)	**0.027**	4.13 (3.98, 4.60)	3.81 (3.22, 4.06)	**0.032**	4.34 (4.04, 4.61)	3.98 (3.37, 4.22)	**0.046**
CLR, M (Q_1_, Q_3_)	1.89 (0.62, 3.07)	1.11 (0.30, 1.31)	0.084	1.32 (0.91, 2.75)	0.38 (0.11, 1.16)	**0.008**	1.31 (0.81, 2.78)	0.91 (0.12, 1.32)	**0.031**	2.75 (1.67, 3.44)	1.05 (0.12, 1.31)	**0.001**

*Note:* The bold values highlight statistically significant results (*p* < 0.05).

Abbreviations: M, median; Q_1_, 1st quartile; Q_3_, 3st quartile; SD, standard deviation.

### 3.4 Correlation Between CLR, LAR, and Intratumoral Immune Infiltration

**FIGURE 4:**
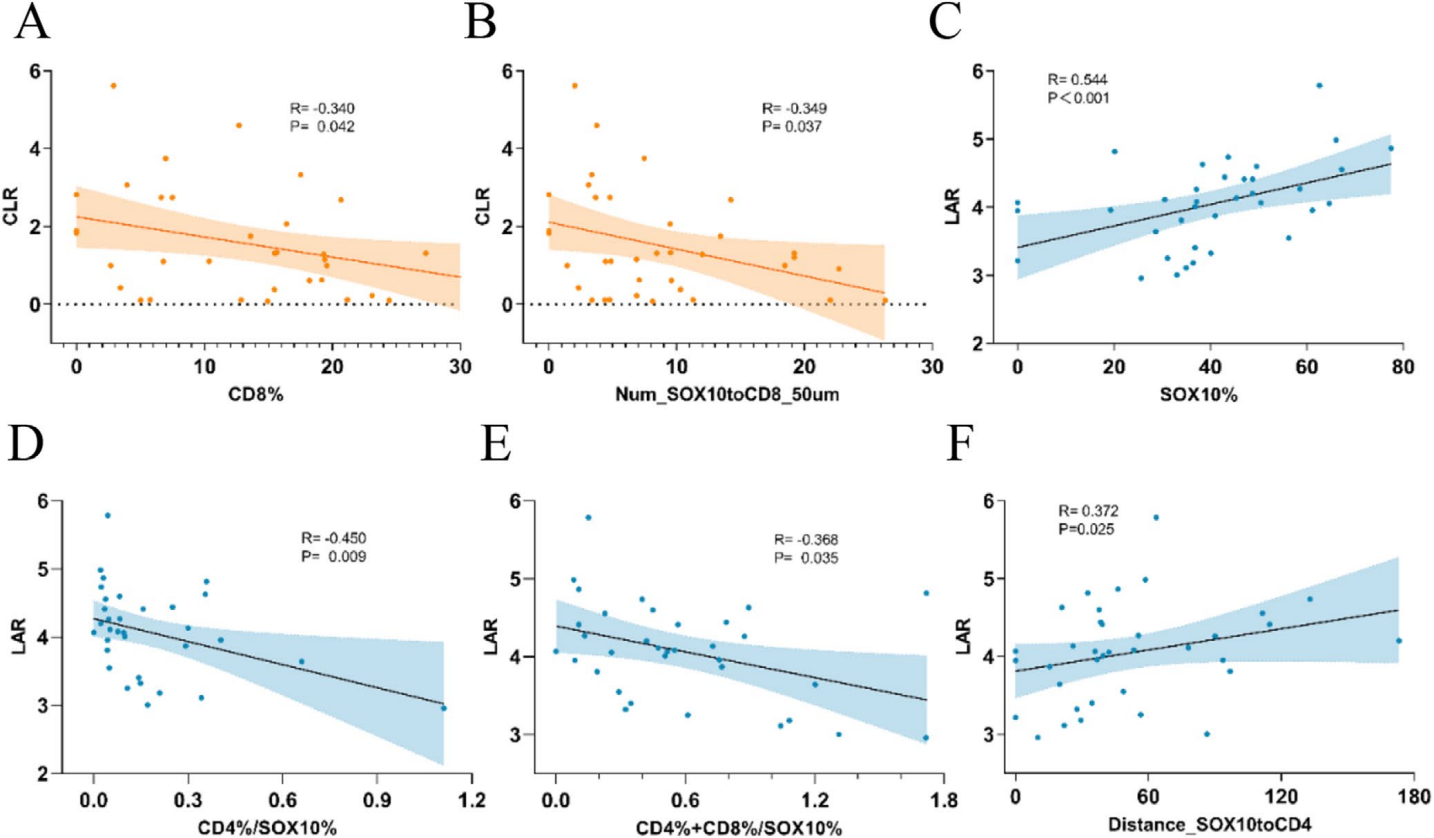
Correlation between blood parameters and TLS status. (A) Correlation between CLR levels and the proportion of CD8^+^ T cells in the TME. (B) Relationship between CLR levels and the number of CD8^+^ T cells within 50 μm of SOX10^+^ tumor cells. (C) Correlation between LAR levels and the proportion of SOX10^+^ tumor cells. (D, E). Correlation between LAR levels and the ratio of CD4^+^ T cells to SOX10^+^ cells, as well as the ratio of (CD4^+^ T + CD8^+^ T) cells to SOX10^+^ cells. (F) Relationship between LAR levels and the distance between CD4^+^ T cells and SOX10^+^ cells.

Considering that tumor‐infiltrating immune cells migrate to tumor sites through the bloodstream, we explored the correlation between immune cell infiltration in TME and blood biomarkers, focusing on the proportions and spatial distribution of immune cell subsets. The results indicated that CLR levels were significantly negatively correlated with the proportion of CD8^+^ T cells in the TME (*R* = −0.34, *p* = 0.042) (Figure 4A), while LAR was positively correlated with the proportion of SOX10^+^ cells (*R* = 0.544, *p* < 0.01) (Figure 4C). Additionally, LAR was negatively correlated with the ratio of CD4^+^ T cells to SOX10^+^ cells, as well as the proportion of (CD4^+^ T + CD8^+^ T) cells (Figure 4D,E). In the cell distance analysis, we observed that CLR levels were positively correlated with the number of CD8^+^ T cells within 50 μm of SOX10^+^ cells, whereas LAR was positively correlated with the distance between SOX10^+^ cells and CD4^+^ T cells (Figure 4B,F). At the same time, we further compared the immune infiltration profiles between patients with poor prognosis in the high CLR and LAR groups and those in the low CLR and LAR groups. The results demonstrated that patients in the high CLR and LAR groups exhibited significantly lower immune infiltration compared to those in the low CLR and LAR groups (Table S2). These findings suggest that elevated CLR and LAR levels are associated with impaired immune infiltration in the TME.

## 4 Discussion

In this study, we performed an in‐depth analysis of clinical factors and hematological parameters to assess their prognostic significance and their association with TLS and immune cell infiltration within the TME. Kaplan–Meier survival curves and multivariate analyses revealed that CLR and LAR levels were significantly associated with overall survival in patients with AM. Moreover, CLR and LAR levels were strongly correlated with the presence of both pTLS and sTLS in these patients. Subsequent analysis of the immune cell composition and spatial distribution within the TME showed a negative correlation between elevated CLR and LAR levels and the degree of immune cell infiltration.

Peripheral blood testing, due to its convenience, repeatability, and low cost, has become a crucial tool for monitoring inflammatory and nutritional biomarkers in patients with various cancers, playing an important role in cancer prognosis assessment. CLR is derived from the ratio of C‐reactive protein (CRP) to lymphocyte count. CRP is a protein synthesized by the liver in response to inflammatory cytokines during conditions such as inflammation, infection, and trauma. It is considered one of the most sensitive biomarkers of inflammation. Elevated CRP levels have been shown to be associated with poor survival rates in various cancers, including renal carcinoma [25], lung cancer [26], pancreatic cancer [27], and breast cancer [28]. Lymphocytes, as key components of the immune system, play a critical role in the body's antitumor immune response. The reduction in lymphocyte count and the elevated CRP levels leading to a lower CRP‐to‐Lymphocyte ratio (CLR) indicate a pro‐tumor inflammatory state and impaired antitumor immune function. Numerous studies have confirmed that elevated CLR levels are closely associated with poor prognosis [29, 30, 31]. Our study is consistent with previous results; CLR > 2.72 was identified as an independent unfavorable prognostic factor for AM patients. Additionally, elevated levels of LDH have been shown to promote tumor progression by modulating tumor metabolism and the tumor microenvironment, and LDH has become an important prognostic marker for poor outcomes in cancer patients [32, 33, 34]. Albumin levels reflect the nutritional status, liver function, and immune defense capacity of the body. A decrease in albumin levels often indicates malnutrition and impaired immune function, which are closely related to cancer onset, progression, and prognosis. Therefore, the LAR (CLR/Alb ratio) provides a comprehensive reflection of the tumor burden, tumor hypoxia, nutritional status, and systemic inflammatory response in patients. Previous research has shown that LAR is strongly associated with the prognosis of patients with different types of cancer and could act as an independent predictor of poor outcomes [14, 35, 36]. In this study, we provide preliminary evidence that LAR > 3.73 can serve as an independent unfavorable prognostic factor for AM patients.

TLS serve as significant immune markers within TME and have been extensively studied across various cancer types. The formation and maturation of TLS are closely linked to the inflammatory and metabolic milieu. Chronic inflammation induced by tumors can drive the ectopic formation of lymphoid tissue, further promoting the development of TLS in both intratumoral and peritumoral areas. Numerous studies have emphasized the prognostic significance of mature TLS in cancer. For example, a study involving non‐small cell lung cancer (NSCLC) patients demonstrated that those with mature TLS exhibited a significantly lower risk of recurrence [37]. Similarly, a cohort study of esophageal squamous cell carcinoma patients showed that individuals with mature TLS exhibited markedly better survival outcomes compared to those with immature TLS or TLS‐negative status [38]. Building on our previous identification of a correlation between CLR and LAR levels and patient prognosis, we further explored whether these markers are associated with the TLS status within TME of cancer patients. Our results indicate that patients without detectable intratumoral TLS, or lacking both intratumoral pTLS and sTLS, exhibit significantly elevated levels of CLR and LAR. In the eTLS group, CLR and LAR levels were also elevated, although this difference did not reach statistical significance. Stratifying patients by TLS maturation stages revealed a significant decrease in CLR and LAR levels with increasing TLS maturity. Moreover, the location of TLS within the tumor has been shown to influence prognosis [39]. However, since nearly all patients exhibited peritumoral TLS, we were unable to compare the levels of CLR and LAR between peritumoral and intratumoral TLS. In conclusion, our study confirms that the hematological markers CLR and LAR reflect the maturity status of intratumoral TLS in AM patients.

The study of TLS can be significantly influenced by sample type and methodology. Our analysis utilized FFPE tissue blocks derived from resected melanoma specimens. Compared with core or shave biopsies, resected specimens provide more comprehensive tissue architecture information, offering a more accurate representation of the tumor microenvironment and facilitating detailed TLS characterization. TLS analysis integrated H&E staining with mIF. Vanhersecke et al. [40] demonstrated that mIF, in combination with H&E and dual CD20/CD23 staining, represents the most sensitive approach for TLS detection. Additionally, melanocytes in tumor tissue often necessitate removal during immunohistochemistry (IHC) staining, a process that can lead to false‐negative results. In contrast, mIF is unaffected by melanin interference, thereby enabling more precise identification of tumor boundaries. Griss et al. [41] corroborated this through MIF analysis of 41 melanoma patients, revealing that tumor‐associated B cells are predominantly localized at the invasive tumor margins [42], thereby underscoring the potential role of B cells in peripheral TLS formation. In our study, intratumoral TLS was observed in 66.67% (24/36) of cases, whereas peritumoral TLS exhibited a higher prevalence at 83.33% (30/36). Lynch et al. [43] identified TLS in 30 of 64 melanoma metastasis patients (47%) using tissue microarray‐based mIF on smaller tissue volumes. Vanhersecke et al. [40] reported that primary tumor samples are twice as likely as metastatic samples to harbor detectable TLS. Similarly, Lee et al. [44] observed a greater abundance of tumor infiltrating lymphocytes in primary tumors (e.g., liver, brain, ovary) compared to matched metastases. The application of whole‐slide mIF on primary melanoma resection specimens in our study may account for the higher TLS detection rates compared to those reported by Lynch et al. However, it is important to note that our study's relatively small sample size (*n* = 36) may introduce bias, and thus, the findings warrant validation in larger‐scale studies, particularly when comparing resection specimens with biopsy samples.

The composition of different immune cell types within TME and their spatial relationship with tumor cells significantly impact tumor growth, metastasis, and prognosis [45, 46, 47, 48, 49]. In pancreatic cancer, specific T cell subsets and cytotoxic T cell density within a 20 μm radius of tumor cells are closely associated with patient survival [50]. In colorectal cancer patients with liver metastasis, prolonged overall survival is associated with the distribution of T cells in the tumor periphery (≤ 10 μm) [51]. Additionally, studies on advanced melanoma have demonstrated that the density of immune cells within a 20 μm radius of melanoma cells is significantly correlated with both the patient's response to immunotherapy and prognosis [52]. Our findings further support these observations. Specifically, in the CLR‐high group, the proportion of CD8^+^ T cells and the number of CD8^+^ T cells surrounding SOX10^+^ cells within a 50 μm radius were significantly reduced, and both parameters exhibited a negative correlation with CLR levels. Similarly, the LAR‐high group displayed a less favorable immune infiltration profile, as indicated by the ratio of CD4^+^ T cells to SOX10^+^ cells and the distance between SOX10^+^ cells and CD4^+^ T cells.

Several studies have explored the correlation between peripheral blood biomarkers and patient prognosis. In this study, we explored the relationship between CLR, LAR, and overall survival in AM patients, and further examined how these blood markers correlate with TLS status and immune cell profiles in the tumor microenvironment. We found that the poorer prognosis associated with elevated CLR and LAR levels may be related to reduced maturity of intratumoral TLS and impaired immune cell infiltration within the TME. This suggests that both local factors and systemic conditions play a crucial role in shaping TLS and immune cell populations within the tumor microenvironment. These findings provide a theoretical framework for utilizing blood biomarkers as predictive tools for patient outcomes in cancer.

This study has several limitations that should be considered when interpreting the results. First, the relatively small sample size may limit the statistical power to detect significant associations between peripheral blood inflammatory markers, TLS, and immune cell infiltration in the TME. Second, being a single‐center study, it may introduce selection bias and restrict the generalizability of the findings to broader patient populations. Third, postoperative anti‐tumor treatments were not included in the analysis, which could potentially affect the dynamics of peripheral blood inflammatory markers and immune cell infiltration in the TME. Future studies should incorporate treatment data to better understand its impact on these parameters. Finally, this study did not dynamically track changes in peripheral blood inflammatory markers or immune cell infiltration in the TME over time. Longitudinal assessments could offer deeper insights into the temporal relationships and potential causal mechanisms underlying these interactions. Despite these limitations, our findings reveal important associations that warrant further investigation in larger, multi‐center studies with more comprehensive and dynamic assessments.

## Author Contributions

All authors contributed to the study conception and design. The experiments, data analysis, and manuscript drafting were carried out by Donglin Kang, Xinyu Su, and Jiayu Wang. The collection of clinical information and clinical follow‐up were carried out by Donglin Kang, Xinyu Su, Jiayu Wang, Lianjun Zhao, and Rong Huang. All authors provided feedback on previous versions of the manuscript. Zhengyun Zou supervised the manuscript review and editing. All authors approved the final manuscript.

## Ethics Statement

The study adhered to the principles of the Declaration of Helsinki and was approved by the Ethics Committee of Nanjing Drum Tower Hospital (Approval No. 2024‐282‐01). Written informed consent was obtained from all participants prior to their inclusion in the study.

## Conflicts of Interest

The authors declare no conflicts of interest.

## Supporting information


Data S1.

## Data Availability

The datasets generated during and analyzed during the current study are available from the corresponding author on reasonable request.
