# Supplementary material for: C‐Reactive Protein to Lymphocyte Ratio (CLR) and Lactate Dehydrogenase to Albumin Ratio (LAR) as Prognostic Biomarkers in Acral Melanoma: Association With Tertiary Lymphoid Structures and Immune Cell Infiltration
Source: Cancer Med. 2025 Jul 26;14(15):e71078. doi: 10.1002/cam4.71078 (PMC12677929; doi:10.1002/cam4.71078)
Supplement: Supplementary file 1 — Data S1. [file CAM4-14-e71078-s001.docx]

| **Supplementary Table 1. Comparison of clinical characteristics and laboratory parameters at different stages of peritumoral TLS maturity** | | | | | | | | | | | | | |
| --- | --- | --- | --- | --- | --- | --- | --- | --- | --- | --- | --- | --- | --- |
| **Variables** | **Peritumoral eTLS** | |  | **Peritumoral pTLS** | |  | **Peritumoral sTLS** | |  | | **Peritumoral TLS** | |  |
|  | **without(n = 7)** | **with(n = 29)** | **P** | **without(n = 22)** | **with(n = 14)** | **P** | **without(n = 21)** | **with(n = 15)** | | **P** | **without(n = 6)** | **with(n = 30)** | **P** |
| **Age (years), Mean ± SD** | 62.86 ± 16.25 | 66.93 ± 8.34 | 0.541 | 66.14 ± 10.18 | 66.14 ± 10.31 | 0.999 | 66.71 ± 9.45 | 65.33 ± 11.41 | | 0.694 | 64.00 ± 14.31 | 66.57 ± 9.41 | 0.58 |
| **Sex, n(%)** |  |  | 1 |  |  | 1 |  |  | | 0.736 |  |  | 0.662 |
| Male | 3 (42.86) | 14 (48.28) |  | 17 (47.22) | 10 (45.45) |  | 9 (42.86) | 8 (53.33) | |  | 2 (33.33) | 15 (50.00) |  |
| Female | 4 (57.14) | 15 (51.72) |  | 19 (52.78) | 12 (54.55) |  | 12 (57.14) | 7 (46.67) | |  | 4 (66.67) | 15 (50.00) |  |
| **Stage, n(%)** |  |  | 0.063 |  |  | 0.897 |  |  | | 1 |  |  | 0.566 |
| I | 2 (28.57) | 2 (6.90) |  | 4 (11.11) | 2 (9.09) |  | 2 (9.52) | 2 (13.33) | |  | 1 (16.67) | 3 (10.00) |  |
| II | 1 (14.29) | 16 (55.17) |  | 17 (47.22) | 11 (50.00) |  | 10 (47.62) | 7 (46.67) | |  | 2 (33.33) | 15 (50.00) |  |
| III | 4 (57.14) | 11 (37.93) |  | 15 (41.67) | 9 (40.91) |  | 9 (42.86) | 6 (40.00) | |  | 3 (50.00) | 12 (40.00) |  |
| **WBC, M (Q₁, Q₃)** | 4.60 (4.20, 5.00) | 6.00 (4.90, 7.70) | **0.01** | 5.80 (4.77, 6.72) | 6.10 (4.90, 8.00) | 0.217 | 5.80 (4.60, 8.10) | 5.80 (4.95, 6.25) | | 0.923 | 4.60 (4.00, 4.68) | 6.00 (4.93, 7.55) | **0.007** |
| **Lymphocytes (×109/L), M (Q₁, Q₃)** | 1.20 (1.15, 1.50) | 1.80 (1.60, 2.00) | **0.006** | 1.75 (1.48, 1.92) | 1.75 (1.42, 2.00) | 0.909 | 1.80 (1.40, 2.00) | 1.70 (1.55, 1.85) | | 0.675 | 1.35 (1.12, 1.50) | 1.80 (1.60, 2.00) | **0.004** |
| **CRP(mg/L), M (Q₁, Q₃)** | 3.10 (1.20, 4.55) | 2.10 (0.80, 3.10) | 0.387 | 2.15 (0.75, 3.32) | 2.20 (1.85, 3.10) | 0.535 | 2.20 (1.10, 3.10) | 2.10 (0.20, 3.90) | | 0.687 | 2.65 (2.05, 4.22) | 2.10 (0.65, 3.25) | 0.405 |
| **LDH(U/L), M (Q₁, Q₃)** | 166.00 (129.00, 180.00) | 169.00 (151.00, 177.00) | 0.576 | 167.50 (148.00, 177.25) | 169.50 (157.00, 177.75) | 0.338 | 174.00 (165.00, 179.00) | 149.00 (130.00, 170.50) | | **0.023** | 169.50 (140.50, 183.50) | 167.50 (149.50, 176.75) | 0.915 |
| **Albumins(g/L), M (Q₁, Q₃)** | 39.60 (39.45, 41.45) | 40.60 (39.20, 41.60) | 0.734 | 40.60 (39.35, 41.70) | 40.25 (38.88, 42.08) | 0.871 | 40.80 (39.40, 42.10) | 40.30 (39.30, 40.75) | | 0.431 | 40.70 (39.77, 41.78) | 40.45 (39.25, 41.53) | 0.899 |
| **LAR, M (Q₁, Q₃)** | 4.07 (3.27, 4.50) | 4.06 (3.81, 4.41) | 0.936 | 4.07 (3.62, 4.42) | 4.09 (3.89, 4.59) | 0.236 | 4.20 (3.96, 4.55) | 3.64 (3.29, 4.10) | | **0.037** | 4.26 (3.46, 4.53) | 4.06 (3.68, 4.38) | 0.656 |
| **CLR, M (Q₁, Q₃)** | 2.82 (1.03, 3.41) | 1.11 (0.43, 1.75) | 0.124 | 1.18 (0.42, 2.22) | 1.22 (0.93, 2.01) | 0.615 | 1.11 (0.62, 2.07) | 1.32 (0.12, 2.32) | 0.923 | | 2.33 (1.46, 3.00) | 1.10 (0.39, 1.85) | 0.071 |

| **Supplementary Table 2. Immune Infiltration Profiles Based on Blood Marker Grouping** | | | | | | |
| --- | --- | --- | --- | --- | --- | --- |
| **Variables** | **CLR-low (n = 28)** | **CLR-high (n = 8)** | **P** | **LAR-low (n = 10)** | **LAR-high (n = 26)** | **P** |
|  |  |  |  |  |  |  |
| **CD4%, M (Q₁, Q₃)** | 4.45 (1.75, 8.52) | 2.82 (2.25, 4.96) | 0.262 | 5.81 (3.80, 10.86) | 2.82 (1.60, 6.97) | 0.1 |
| **CD8%, M (Q₁, Q₃)** | 16.04 (9.47, 20.80) | 6.80 (3.69, 8.79) | **0.021** | 15.49 (9.02, 22.25) | 13.91 (5.20, 19.38) | 0.448 |
| **CD68%, M (Q₁, Q₃)** | 13.14 (5.96, 23.31) | 11.81 (2.41, 23.29) | 0.805 | 13.21 (9.36, 19.34) | 11.52 (3.69, 25.05) | 0.818 |
| **SOX10%, M (Q₁, Q₃)** | 37.69 (30.93, 48.71) | 49.31 (37.02, 63.46) | 0.189 | 33.99 (29.26, 36.68) | 44.51 (36.86, 56.51) | **0.025** |
| **CD4%/SOX10%, M (Q₁, Q₃)** | 0.10 (0.05, 0.30) | 0.06 (0.03, 0.10) | 0.058 | 0.17 (0.14, 0.34) | 0.08 (0.04, 0.16) | **0.014** |
| **CD8%/SOX10%, M (Q₁, Q₃)** | 0.42 (0.36, 0.54) | 0.14 (0.06, 0.19) | **0.004** | 0.54 (0.24, 0.70) | 0.38 (0.11, 0.43) | **0.033** |
| **CD4%+CD8%/SOX10%, M (Q₁, Q₃)** | 0.59 (0.40, 0.89) | 0.19 (0.12, 0.33) | **0.003** | 1.04 (0.35, 1.20) | 0.45 (0.15, 0.73) | **0.03** |
| **Num CD68toCD8 (20µm), M (Q₁, Q₃)** | 3.07 (2.24, 5.28) | 1.69 (1.07, 2.22) | **0.046** | 3.05 (2.08, 5.74) | 2.66 (1.50, 3.89) | 0.513 |
| **Num CD68toCD8 (50µm), M (Q₁, Q₃)** | 12.24 (9.16, 19.23) | 5.18 (3.28, 9.80) | **0.046** | 13.48 (9.47, 23.71) | 10.03 (4.91, 16.57) | 0.197 |
| **Distance CD68toCD8 (µm), M (Q₁, Q₃)** | 11.31 (7.82, 16.53) | 15.38 (10.49, 22.35) | 0.351 | 11.04 (6.37, 15.42) | 11.69 (9.25, 20.35) | 0.406 |
| **Num SOX10toCD4 (20µm), M (Q₁, Q₃)** | 0.59 (0.43, 1.09) | 0.50 (0.39, 0.80) | 0.505 | 0.83 (0.60, 1.71) | 0.49 (0.32, 0.92) | 0.074 |
| **Num SOX10toCD4 (50µm), M (Q₁, Q₃)** | 2.42 (1.39, 4.46) | 2.37 (1.47, 3.81) | 0.689 | 3.44 (2.52, 8.82) | 1.99 (1.28, 3.91) | 0.05 |
| **Num SOX10toCD8 (20µm), M (Q₁, Q₃)** | 1.68 (0.91, 2.42) | 0.94 (0.56, 1.14) | 0.05 | 1.68 (1.57, 3.59) | 1.51 (0.75, 2.05) | 0.124 |
| **Num SOX10toCD8 (50µm), M (Q₁, Q₃)** | 8.96 (4.68, 13.62) | 3.51 (2.84, 4.00) | **0.008** | 9.92 (7.71, 19.87) | 4.81 (3.35, 9.58) | **0.042** |
